# Identification of TBK1 inhibitors against breast cancer using a computational approach supported by machine learning

**DOI:** 10.3389/fphar.2024.1342392

**Published:** 2024-03-19

**Authors:** Arif Jamal Siddiqui, Arshad Jamal, Mubashir Zafar, Sadaf Jahan

**Affiliations:** ^1^ Department of Biology, College of Science, University of Ha’il, Ha’il, Saudi Arabia; ^2^ Department of Family and Community Medicine, College of Medicine, University of Ha’il, Ha’il, Saudi Arabia; ^3^ Department of Medical Laboratory Sciences, College of Applied Medical Sciences, Majmaah University, Al Majmaah, Saudi Arabia

**Keywords:** TBK1 inhibitor, binding energy, machine learning, breast cancer, molecular dynamics, hydrogen bonding

## Abstract

**Introduction:** The cytosolic Ser/Thr kinase TBK1 is of utmost importance in facilitating signals that facilitate tumor migration and growth. TBK1-related signaling plays important role in tumor progression, and there is need to work on new methods and workflows to identify new molecules for potential treatments for TBK1-affecting oncologies such as breast cancer.

**Methods:** Here, we propose the machine learning assisted computational drug discovery approach to identify TBK1 inhibitors. Through our computational ML-integrated approach, we identified four novel inhibitors that could be used as new hit molecules for TBK1 inhibition.

**Results and Discussion:** All these four molecules displayed solvent based free energy values of −48.78, −47.56, −46.78 and −45.47 Kcal/mol and glide docking score of −10.4, −9.84, −10.03, −10.06 Kcal/mol respectively. The molecules displayed highly stable RMSD plots, hydrogen bond patterns and MMPBSA score close to or higher than BX795 molecule. In future, all these compounds can be further refined or validated by *in vitro* as well as *in vivo* activity. Also, we have found two novel groups that have the potential to be utilized in a fragment-based design strategy for the discovery and development of novel inhibitors targeting TBK1. Our method for identifying small molecule inhibitors can be used to make fundamental advances in drug design methods for the TBK1 protein which will further help to reduce breast cancer incidence.

## 1 Introduction

**FIGURE 1:**
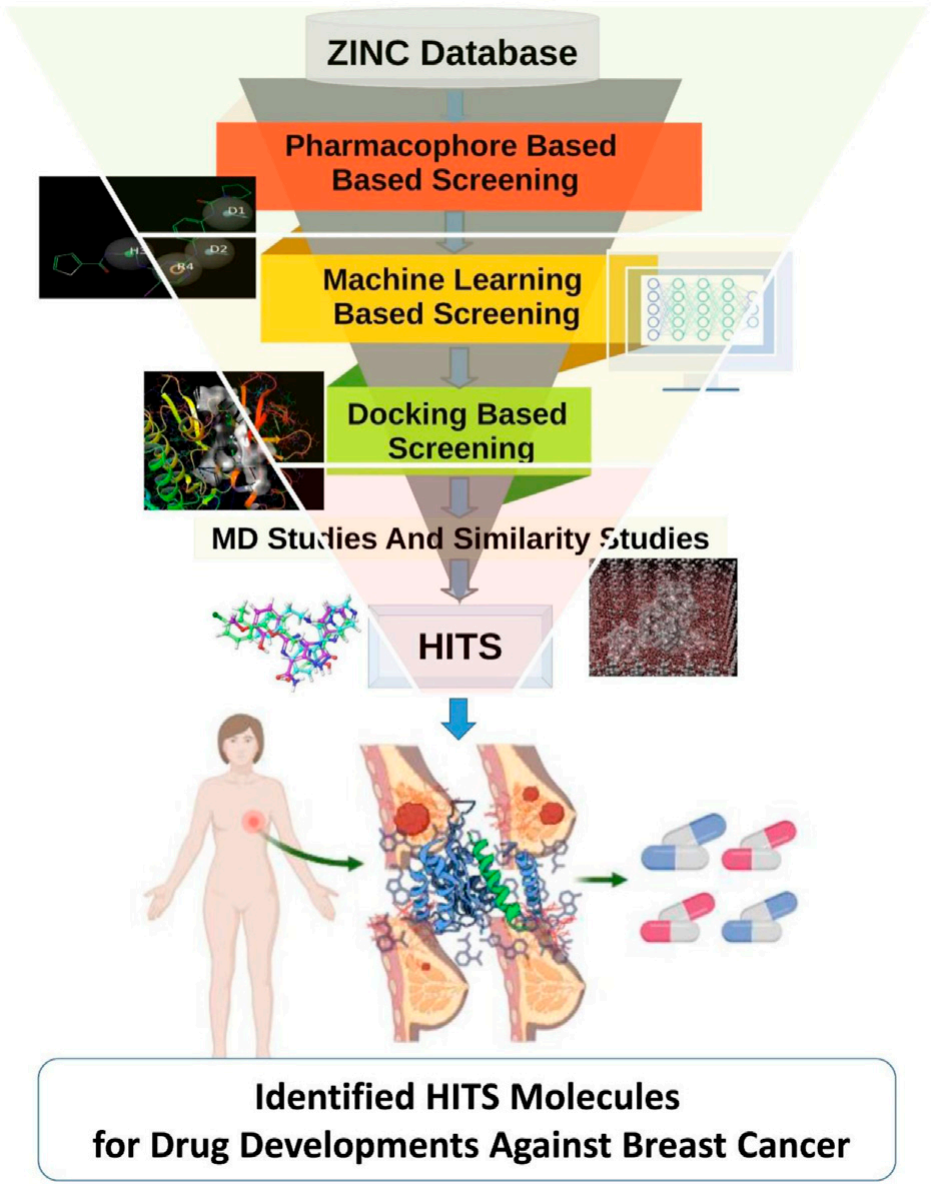
The image indicated the workflow employed in the studies.

TANK-binding kinase 1 (TBK1) stands as a significant player in the intricate network of cellular signaling pathways, exerting its influence through phosphorylation events primarily on serine and threonine residues (Runde et al., 2022). Its functional repertoire spans a wide spectrum of physiological processes, ranging from innate immune responses to cellular homeostasis and beyond. Through intricate interactions with various cellular components, TBK1 orchestrates the delicate balance required for proper immune function and cellular integrity. At the forefront of TBK1’s roles lies its involvement in innate immunity. Through its coordination of critical transcription factors such as interferon regulatory factors (IRFs) and nuclear factor kappa B (NF-κB), TBK1 plays a pivotal role in regulating immune responses against invading pathogens (Tang et al., 2021). Upon detection of viral infections, TBK1-mediated signaling cascades culminate in the synthesis of type I interferons (IFNs) and other cytokines with potent antiviral properties (Siddiqui et al., 2020; Zhang et al., 2021). This orchestrated response not only aids in combating viral infections but also assumes a central role in the broader antiviral defense mechanism of the host organism. Furthermore, TBK1’s influence extends beyond the realm of immune responses, encompassing essential cellular processes such as autophagy and mitochondrial quality control (Pied et al., 2022). Its association with autophagy underscores its role in the degradation and recycling of impaired cellular constituents, thereby contributing to cellular homeostasis and survival (Xiao et al., 2017). The dysregulation of TBK1 has emerged as a significant contributing factor in various pathological conditions, including autoimmune disorders, neurological diseases, and cancer (Siddiqui et al., 2022). In the context of cancer, TBK1’s multifaceted roles present a complex landscape wherein it can exhibit both oncogenic and tumor-suppressive properties. Studies have implicated TBK1 in facilitating cell survival, proliferation, and resistance to apoptosis, mechanisms that are inherently associated with cancer progression (Durand et al., 2018). TBK1 has garnered significant attention from researchers due to its involvement in various biological processes such as inflammation, cellular signaling, and immunological responses (Hu et al., 2020). Notably, TBK1’s involvement in survival pathways has been implicated in lung cancer and specific subtypes of breast cancer, underscoring its relevance in the context of tumorigenesis and tumor progression. Moreover, the interplay between TBK1 and the inflammatory process within the tumor microenvironment further highlights its significance in cancer biology (Xu et al., 2018). Multiple studies indicate that TBK1 has a role in cancer by facilitating cell survival, proliferation, and resistance to apoptosis, which is the programmed cell death process (Revach et al., 2020; Alam et al., 2021; Runde et al., 2022). TBK1 has been implicated in the facilitation of survival pathways in lung cancer, as well as the promotion of growth in specific subtypes of breast cancer (Hasan et al., 2017). Chronic inflammation has long been recognized as a hallmark of cancer, fostering a microenvironment conducive to tumorigenesis and disease progression. TBK1’s participation in the inflammatory response not only underscores its multifaceted roles but also presents potential avenues for therapeutic intervention in cancer (Cruz and Brekken, 2018; Yan et al., 2023). TBK1 has garnered significant attention as a prospective therapeutic target due to its role in signaling pathways associated with cancer. TBK1 influence the cellular milieu and potentially impacting cancer progression. The convergence of TBK1’s roles in immune responses, inflammation, and cancer underscores its potential as a promising therapeutic target for cancer treatment. Despite the recognition of TBK1’s significance, therapeutic interventions targeting TBK1 remain a challenge. Existing inhibitors such as BX795 and CYT387, while potent, face limitations due to specificity issues, underscoring the need for novel and more refined TBK1 inhibitors (Feldman et al., 2005; Pardanani et al., 2009; Sun et al., 2022). In this context, computational methods offer a promising avenue for identifying potential TBK1 inhibitors with improved specificity and efficacy. The field of Computer-Aided Drug Design (CADD) employs computational techniques to facilitate the search, prediction, and identification of small compounds (known as ligands) that exhibit the capability to interact with a designated target molecule. This target molecule is often a disease-associated protein (Sliwoski et al., 2014; Yu and MacKerell, 2017; Siddiqui et al., 2021). The field of CADD has emerged as a valuable tool in drug discovery, facilitating the search, prediction, and identification of small molecules capable of interacting with target proteins. Through virtual screening and computational modeling approaches, researchers can efficiently navigate vast chemical databases to identify lead compounds with the potential for therapeutic application (Schneider and Fechner, 2005; Torres et al., 2019; Sharma et al., 2020; Sahakyan, 2021; Shen et al., 2021; Siddiqui et al., 2023a; Siddiqui et al., 2023b). The primary objective of our present investigation was to develop a computational approach augmented with machine learning techniques in order to find candidate compounds that have the potential to be utilized for *in vitro* validation, specifically targeting the TBK1 protein. In the present study, the researchers utilized the extensive ZINC database to employ machine learning techniques in the process of screening for prospective lead compounds. Our work tries to add to the growing field of precision medicine by bridging the gap between computational methods and cancer therapies. By finding new TBK1 inhibitors, we hope to make it possible for more targeted and effective breast cancer treatments, which will eventually lead to better patient outcomes and quality of life. The methodology utilized in our research has been depicted in Figure 1.

## 2 Materials and methods

### 2.1 Data collection and preparation

In this study, the ZINC database was employed to discover inhibitors in our method targeting the TBK1 protein. The ZINC database was acquired from the web server in the form of SMILES format and subsequently transformed into SDF format using the Open Babel tool (O'Boyle et al., 2011; Irwin et al., 2020). ZINC database had over 20 million molecules. In order to build a machine learning model, obtaining drug activity data against the TBK1 protein was essential. To achieve this, researchers accessed the well-documented ChEMBL database. Specifically, data related to *Homo sapiens* was retrieved in CSV format, and data processing was performed using the pandas library in Python programming language. The Rapid Decoy Retriever (RADER) was employed to achieve data balance by constructing decoys (Wang et al., 2017).

### 2.2 Pharmacophore model generation and virtual screening

The RCSB-PDB database has comprehensive data pertaining to the inhibitory activity of several inhibitors on the TBK1 protein, including details regarding their respective bound conformations. The phase module of the Schrodinger software was employed for the generation of a multi-template pharmacophore (Dixon et al., 2006b). Pharmacophore structures were generated by extracting conformations of established inhibitory drugs from the Protein Data Bank (PDB) database. The creation of flexible molecular aligned pharmacophore characteristics was achieved through the process of superimposing and complying to similarity constraints. The conformational data incorporated the angles of rotation of the flexible bonds. The software generates molecular conformations that fulfill the alignment of molecules. The assessment of alignment fitness involves evaluating the similarity and quantity of aligned features, the volume of overlapping features, and the Van der Waals energy of the conformation. Next, the pharmacophoric features are extracted, and subsequently, angle and distance constraints are incorporated. Additional investigations using MD (Molecular Dynamics) were incorporated into our methods to ascertain the crucial residues implicated in interactions and their contribution to ligand stability. The conclusions drawn from these research were utilized to construct a pharmacophore model based on ligand structure. The generated pharmacophore was employed to conduct a screening of the ZINC database in order to discover a list of compounds that exhibit pharmacophore properties similar to those of previously described TBK1 inhibitors (Dixon et al., 2006a). The compounds that passed the initial selection process were stored in the ZNHT database for subsequent computational screening procedures.

### 2.3 Descriptor calculation

“Mordred”, a python based tool was used for generating different descriptors (Moriwaki and Tian, 2018). Mordred is a versatile molecular descriptor calculation software, offering over 1800 descriptors in both 2D and 3D formats. It provides fast performance, easy installation, and broad platform compatibility, making it an ideal choice for cheminformatics studies, with benchmark results showcasing superior speed compared to PaDEL-Descriptor. It uses detour matrix algorithm for generating descriptors and is twice as fast as other reported methods. This algorithm divide all points of articulation of chemical structures into subparagraphs. Following that, each subgraph’s detour matrix is computed. Finally, further entries are filled and the subgraph detour matrices are merged. It calculated an array of different descriptors for our activity data including 1d, 2d, 3d and various fingerprint based descriptors including pubchem, MACCS, GraphOnly for our datasets.

### 2.4 Model generation and evaluation

There has been a substantial research published about the utilization of machine learning (ML) techniques within the field of computational drug development. The Scikit-learn machine learning package was employed in this study, utilizing Python version 3.10 (Van Rossum and Drake, 2009; Uddin et al., 2022). The dataset was partitioned into training and testing sets using the random shuffle and train test split module provided by scikit-learn. The dataset was partitioned into training and testing sets using an incremental approach, gradually adjusting the train-test ratio from 80/20 to 70/30. This step was taken to optimize the model and achieve the highest possible accuracy. A total of 29 distinct models were assessed in order to determine the optimal model for our dataset based on a range of statistical criteria. The evaluation encompassed a variety of models, namely, XGBoost, Random Forest, Multiple Layer Perceptron, Support Vector Machine (SVM), Linear SVM, Decision Tree, Logistic Regression, Gaussian Naive Bayes, and Extra Tree Classifier. The statistical metrics included in this study included accuracy, receiver operating characteristic (ROC), area under the curve (AUC), and F1 Score for each model. The highest-performing models were employed to evaluate the results acquired from the pharmacophore-based screening of the ZINC database, which was saved as the ZNHT database.

### 2.5 ML based virtual screening of ZNHT database

The best machine-learning model was used to screen the ZNHT database created after screening the ZINC database. After this dual screening approach we were able to shortlist numerous molecules having both pharmacophore based profile as well as ML based signature pattern. Thus, the shortlisted hits were then subjected to more robust docking based computational screening method.

### 2.6 Molecular docking and interaction studies

The crystal structure of TBK1 bound to the BX795 inhibitor (PDB ID 4IM2) was used for docking studies (Tu et al., 2013; Rigden and Fernández, 2021). Docking studies were performed using the Glide docking module (Friesner et al., 2006). Hydrogen atoms were incorporated using the Protein Preparation Wizard and subsequent energy minimization was conducted to mitigate steric clashes of the protein, utilizing the Maestro interface within the Schrödinger Suite. The resulting prepared structure served as the basis for subsequent docking studies. Ligands molecules were cleaned using ligprep module. Different parameters which were optimized include ligand sampling, ring flexibility for ligands, docking poses for post dock minimization, inclusion of partial charges in scoring scheme. 20 poses were retained for each ligand to perform post dock minimization, while only top 3 poses were retained in final results.

### 2.7 Molecular dynamics simulation

In the pursuit of identifying new inhibitors, molecular dynamics (MD) simulations prove to be a valuable tool as they enable the assessment of the ligand’s stability within the binding pocket of the receptor. The MD simulations using Gromacs 2022.05 as stated in references (Van Der Spoel et al., 2005; Siddiqui et al., 2023c). The CHARMM parameters were obtained using the SwissParam web service (Zoete et al., 2011). The previous publications provide a comprehensive description of the methodology utilized for establishing simulation studies in GROMACS (Siddiqui et al., 2023b). The examination of the molecular dynamics (MD) findings was conducted utilizing the UCSF-Chimera program (Pettersen et al., 2004). The plots were generated utilizing the XMGRACE software package [https://plasma-gate.weizmann.ac.il/Grace/], while the calculations relying on MMPBSA were conducted employing the Amber software (Valdés-Tresanco et al., 2021).

### 2.8 Similarity index studies and physiochemical properties studies

Database searches for molecules that resemble specific structures have grown in popularity over the past 10 years. Cheminformatics, chemistry, and pattern recognition are just a few of the applications and domains that make use of the similarity searching notion (Rácz et al., 2018). Due to the rising demand for drug discovery research, molecular searching has recently emerged as one of the major themes of cheminformatics study. Here in our studies we have used RDKit to calculate molecular similarity based on Tanimoto coefficient between two lists of molecules with SMILE structures [Landrum, 2010. “RDKit.” Q2. https://www.rdkit.org/.] (Landrum, 2010). The RDKit module was used using python library. Physiochemical properties including LogP, molecular weight, number of hydrogen bond donors and acceptors were also calculated using the RDKIT package. ADMET properties were calculated using the SwissADME web server (Daina et al., 2017).

## 3 Results and discussion

### 3.1 Data collection and preparation

The activity data pertaining to the compounds’ inhibition of TBK1 was obtained by downloading the information from the CHEMBL database, specifically identified by the unique identifier “CHEMBL5408.” The dataset was assessed for redundancy, and any duplicate molecules were eliminated. The investigation revealed a broad spectrum of molecular activity, ranging from 0.06 nM to 50118723.36 nM, thereby presenting a wide array of inhibitors exhibiting varying levels of activity. In our binary categorization approach, compounds were categorized as active molecules if their IC50 values were less than 1 μM, while compounds with IC50 values more than 1 µM were considered inactive. By utilizing the aforementioned criteria, a cumulative count of 629 molecules exhibiting proven activity was obtained, while 164 molecules were identified as confirmed inactive. Due to an imbalance in the quantity of active and inactive molecules, the model constructed using the aforementioned dataset may exhibit a bias towards the identification of active molecules. In order to achieve data balance, decoys were generated from the RADER method, resulting in the addition of 469 molecules to the inactive dataset. Following the process of data balancing, we observed a near-equilibrium ratio of approximately 1:1 between active and inactive molecules. After the inclusion of decoy molecules, the dataset had a total of 1,262 molecules. By removing the decoy, we obtained a dataset that achieved balance in terms of representing both classes. This balanced dataset may now be utilized to construct a machine learning model that is free from bias.

### 3.2 Pharmacophore model generation and virtual screening

**FIGURE 2:**
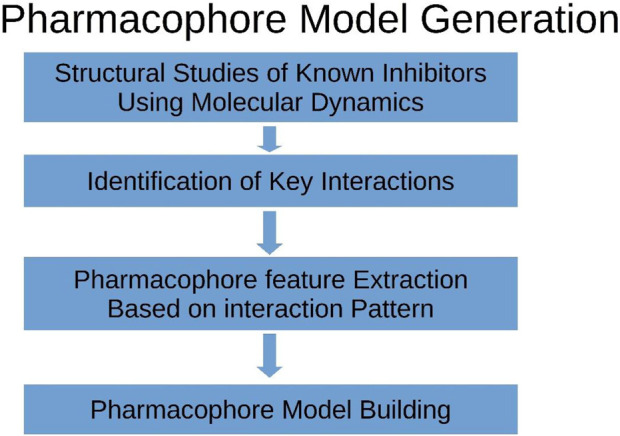
The graphic provides an explanation of the procedure that was followed when developing the pharmacophore model.

The RCSB-PDB database encompasses a diverse array of compounds, each accompanied by their respective inhibitory properties and associated binding complexes involving TBK1 (Beyett et al., 2018). The methodology employed in our experiments for constructing the pharmacophore model was based on the framework depicted in Figure 2. The bound conformation of this chemical was utilized in the development of a structure-ligand-based pharmacophore hypothesis. The pharmacophore hypothesis was developed using the phase module of Schrodinger, which involved extracting group features from the reported conformation of known ligands.

The RCSB-PDB database contains a wide range of chemicals, each with their corresponding inhibitory characteristics and related binding complexes including TBK1 (Ma et al., 2012). The methodology utilized in our experimental approach for creating the pharmacophore model was derived from the framework illustrated in Figure 2. The utilization of the bound conformation of this chemical played a crucial role in the creation of a structure-ligand-based pharmacophore hypothesis. The pharmacophore hypothesis was constructed utilizing the phase module of Schrodinger software, which entailed collecting group characteristics from the documented conformation of established ligands.

**FIGURE 3:**
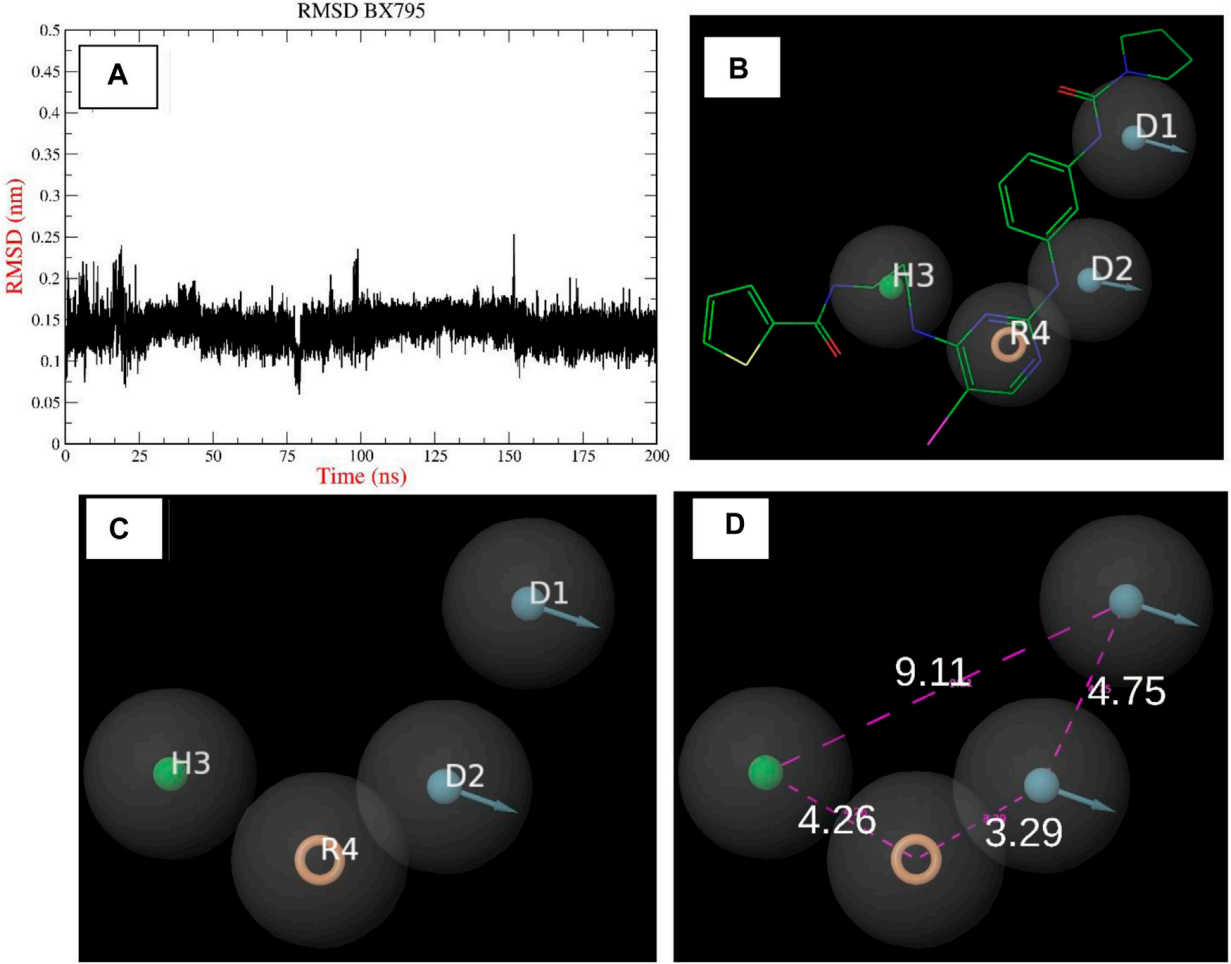
**(A–D)**: The Image indicates the pharmacophore model built using the reported crystal structure of TBK1 inhibitor BX795.

The chemical BX795 has been shown to display TBK1 inhibition at a concentration of 6 nM, as documented in prior studies (Clark et al., 2009). The hydrophobic residues, specifically L15, G16, G18, A21, A36, M86, M142, T156, and V168, as well as the charged or uncharged group residues, notably Q17, K38, F88, N140, and D157, were identified within a 5 Å proximity of BX795. The presence of a positively charged residue, specifically K38, was observed to engage in cation-pi interactions with the receptor. Therefore, the utilization of an aromatic ring in the pharmacophore has the ability to replicate this interaction. In a manner akin to the aforementioned scenario, the nitrogen atom in proximity to the aromatic ring engaged in an interaction with the N140 residue, assuming the role of a donor group. The selection of a hydrophobic attribute was based on the observation that the hydrophobic residues were predominantly located in close proximity to the formamido-propyl group in the control molecule. The construction of a 4-feature pharmacophore model, was facilitated by conducting simulation studies on control molecules and interaction experiments on established ligands as depicted in Figures 3A–D. This model incorporates 1 hydrophobic feature (H3), 2 donor groups (D1 and D2), and a single aromatic group (R4).

**FIGURE 4:**
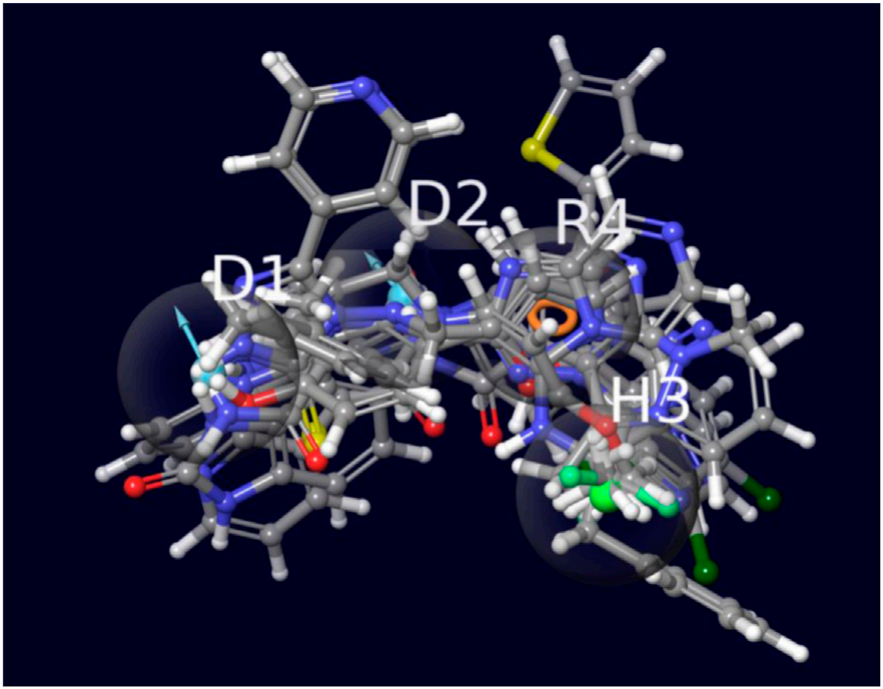
The image indicates the top selected molecules overlapped with the 4 feature pharmacophore model.

The elongation of the hydrophobic pocket was noticed, prompting the inclusion of an additional 15% tolerance to this characteristic. Given the significance of donor groups in facilitating hydrogen bond interactions, we incorporated a 15% increase in weightage for these groups. This approach would enable the selection of hits that possess these specific groups, which can play a significant role in protein interaction. In order to establish distance limits between the various pharmacophore groups, a tolerance range of 10%–15% was implemented. Following the implementation of the aforementioned model, a virtual screening of the ZINC database was conducted, resulting in the identification of 132,571 hit molecules. This screening was performed on a comprehensive database consisting of around 2 million compounds. The screen’s saved hits, stored in the ZNHT database, accounted for approximately 0.66% of the total hits from the original database (Figure 4). During the initial filtration process, about 99.33% of the compounds were eliminated due to the absence of pharmacophore characteristics that corresponded to previously identified recognized inhibitors.

### 3.3 Descriptor calculation

The “Mordred” python tool was utilized to derive several descriptors for the dataset that was obtained by downloading. Various descriptors were computed for our dataset, including 1D, 2D, and 3D descriptors, as well as fingerprint-based descriptors such as PubChem, MACCS, and Graph. The descriptors underwent preprocessing to identify and correct any mistakes, utilizing the numpy and pandas packages. Following the pre-processing stage, the data was utilized for machine learning modeling.

### 3.4 Model generation and evaluation

**FIGURE 5:**
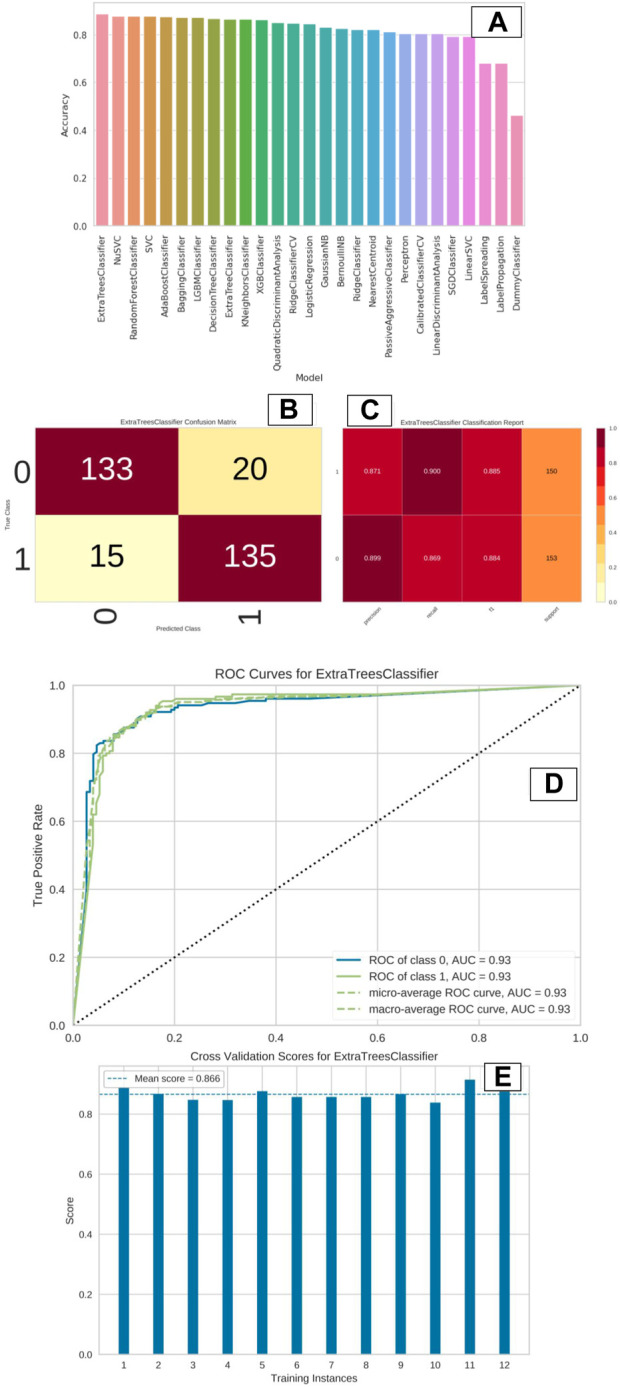
**(A–E)**: The image “**(A)**” indicate the accuracy plot of various ML models represented in the form of vertical bar plot. Image “**(B)**” indicate the confusion matrix of “Extra Trees Classifier” model, which displayed the best accuracy. Image “**(C)**” indicate the classification report of the “Extra Tree Classifier” model in terms of precision, recall, F1 and support values. Image “**(D)**” indicate the ROC curve of “Extra Tree Classifier” model. Image “**(E)**” indicate the cross validation score of “Extra Tree Classifier” model.

The entire dataset is partitioned into training and test sets, with a gradual adjustment of the ratio from 80/20 to 70/30, in order to improve the model and achieve the highest level of accuracy. The training set consisted of a total of 959 compounds, whereas the test set contained 303 compounds, as indicated in Table 1. The molecules classified as active within the dataset were denoted by the label “1,” whilst the molecules classified as inactive were denoted by the label “0.” The dataset was utilized to construct a binary classification model employing a selection of prominent machine learning methods such as random forest, support vector machine, ada boost classifier, extra tree classifier, decision tree classifier, bagging classifier, and others. Out of the 29 algorithms considered, the Extra Trees classifier exhibited the highest levels of accuracy (0.89), ROC (0.88), AUC (0.88), and F1 score (0.89). The NuSVC and random forest classifier closely followed, as depicted in Figure 5A.

**TABLE 1:** Training and test dataset used in our study.

Dataset	Inhibitor	Non-inhibitor	Total
Training	479	480	959
Test	150	153	303

The remaining models had a high degree of correlation, particularly the top four models. The tabulation of top 10 machine learning models based on distinct statistical parameters has been presented in Table 2. The Extra tree classifier model that was chosen as the best performer exhibited notably high levels of accuracy in both the training and test datasets, as evidenced by the confusion matrix depicted in Figure 5B. The model exhibited a high level of precision in accurately identifying inactive chemicals (0.899), closely followed by its performance in identifying active molecules (0.871). The recall value exhibited a very high performance for active compounds (0.900), with inactive molecules closely trailing behind (0.869). Figure 5C demonstrates that both the F1 score and support values were high. The area under the receiver operating characteristic (ROC) curve was determined to be 0.93 for both the active and inactive classes (Figure 5D). The additional tree classifier demonstrated a significantly high cross-validation score of 0.856 when employing a 12-fold cross-validation technique (Figure 5E). The Extra Tree Classifier model was chosen for binary classification of the ZNHT database, which was developed through virtual screening of the ZINC database using a pharmacophore-based technique, depending on the statistical parameters.

**TABLE 2:** The table indicates the ROC, AUC, precision recall and F1 score of top 10 ML models.

Sr No.	Model	Accuracy	ROC	AUC	F1 score
1	ExtraTreesClassifier	0.89	0.88	0.88	0.89
2	NuSVC	0.88	0.87	0.88	0.88
3	RandomForestClassifier	0.88	0.87	0.88	0.88
4	SVC	0.89	0.87	0.88	0.88
5	AdaBoostClassifier	0.87	0.87	0.87	0.87
6	BaggingClassifier	0.87	0.87	0.87	0.87
7	LGBMClassifier	0.87	0.87	0.87	0.87
8	DecisionTreeClassifier	0.87	0.87	0.87	0.87
9	ExtraTreeClassifier	0.86	0.86	0.86	0.86
10	KNeighborsClassifier	0.86	0.86	0.86	0.86

### 3.5 ML based screening of ZNHT database

The ZNHT database, which has a total of 132,571 molecules, was employed in order to ascertain probable hit compounds that could interact with the TBK1 receptor. The descriptors data utilized for constructing the machine learning model was computed for ZNHT molecules employing the Mordred package. Subsequently, the data was stored in a “ csv” format utilizing the pandas data-frame module in Python. The descriptor file was successfully loaded and subsequently utilized to conduct machine learning-based screening of the ZNHT database. A total of 4,350 hits were identified from the ZNHT database, exhibiting fingerprint similarity to the active compounds. Therefore, by utilizing the secondary machine learning-based filter, we successfully excluded around 96.71% of the molecules contained in the ZNHT database. Consequently, we retained a mere 3.28% of hits for further computational investigation.

### 3.6 Molecular docking and interaction studies

**FIGURE 6:**
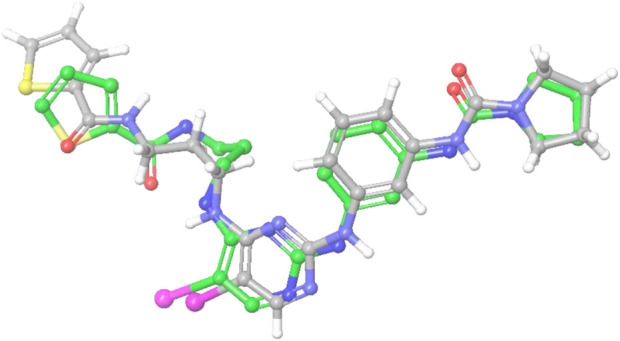
The image indicates the re-dock pose (green) of the control molecule predicted by the Glide docking software compared with the reported crystal pose (grey).

**FIGURE 7:**
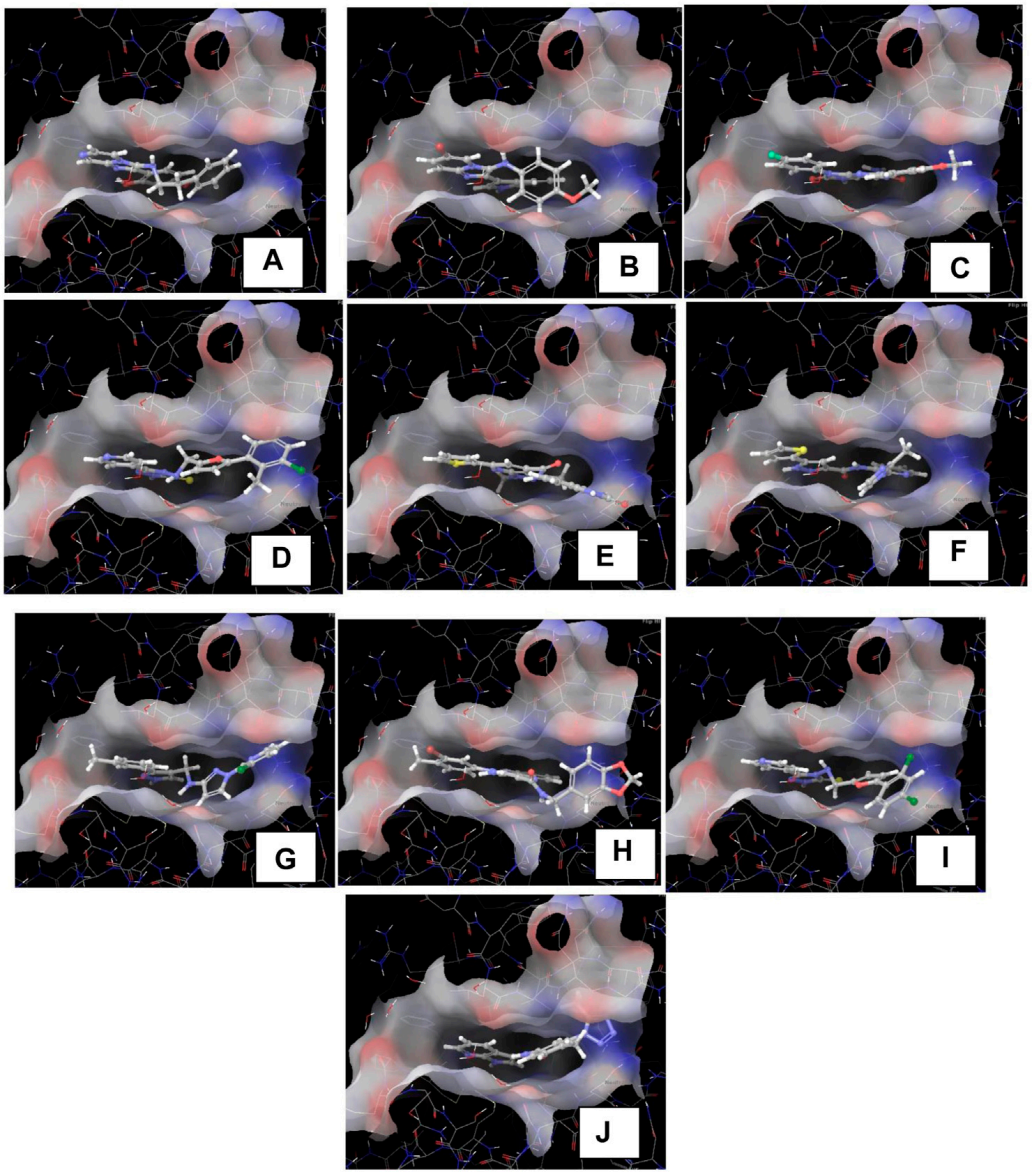
**(A–J)**: The images **(A–J)** indicates the docked poses predicted by the glide docking module of the inhibitors 1–10 respectively.

The crystallographic structure of the TBK1 protein complexed with the antagonist BX795 (PDB id 4IM2) was employed in our investigation of structural binding. This structure served as the basis for conducting docking investigations. The structure was experimentally determined by X-ray diffraction studies. It has resolution of 2.50 Å and R-value of 0.255. The Glide XP docking approach was employed to conduct a docking-based assessment of compounds in order to determine their binding affinity to the receptor. Various parameters were tuned in the glide tool in order to achieve the most optimal redocking stance, as depicted in Figure 6. Following a meticulous optimization of the docking parameters, we successfully generated the docking pose, exhibiting a root-mean-square deviation (RMSD) of less than 0.19 Å. The re-dock position exhibited a binding affinity of −9.641 kcal/mol. The propyl chain of BX795 exhibited modest torsional restrictions, resulting in minor structural aberrations in the thiophenyl ring. After conducting docking-based screening, it was shown that only 10 compounds exhibited a binding affinity higher than the control molecule. The compounds were subjected to thorough analysis in order to assess their potential for molecular interaction with the receptor, as depicted in Figures 7A–J. The observation was made that all compounds exhibited binding to the identical binding pocket of the TBK1 protein, but with diverse binding conformations. Compound 1 exhibited a docking score of −10.4 kcal/mol, while molecule 2 demonstrated a binding affinity of −10.35 kcal/mol, making it a close contender. In a similar vein, molecule 3 had a binding affinity of −10.34 kcal/mol, whilst molecule 4 demonstrated an affinity of −10.04 kcal/mol. The summary of the binding affinities of the compounds that have been nominated is presented in Table 3. The 2d structures of the compounds have been summarized in Supplementary Table S1

**TABLE 3:** The table indicates the Glide docking score and the MMPBSA score summary of the top shortlisted molecules.

Sr No.	Name of compound	Code used	Glide score	MMPBSA score
1	ZINC12113810	1	−10.4	−48.78
2	ZINC12370930	2	−10.35	−12.30
3	ZINC04338236	3	−10.34	−32.50
4	ZINC08548500	4	−10.06	−45.47
5	ZINC26330579	5	−10.03	−46.78
6	ZINC98100095	6	−10.03	−39.83
7	ZINC89797427	7	−9.95	−41.48
8	ZINC02278530	8	−9.91	−42.47
9	ZINC22111451	9	−9.85	−39.07
10	ZINC69924561	10	−9.84	−47.56
11	BX795	CNT	−9.64	−47.11

The molecules underwent molecular dynamics (MD) studies, which will be further elaborated in the subsequent section. All of the compounds exhibited binding to the identical binding pocket of the TBK1 protein. The most significant observation is that all the compounds had a comparable interaction pattern, with a little greater hydrogen bond interaction compared to the control molecule.

### 3.7 Molecular dynamics simulation and free energy studies

**FIGURE 8:**
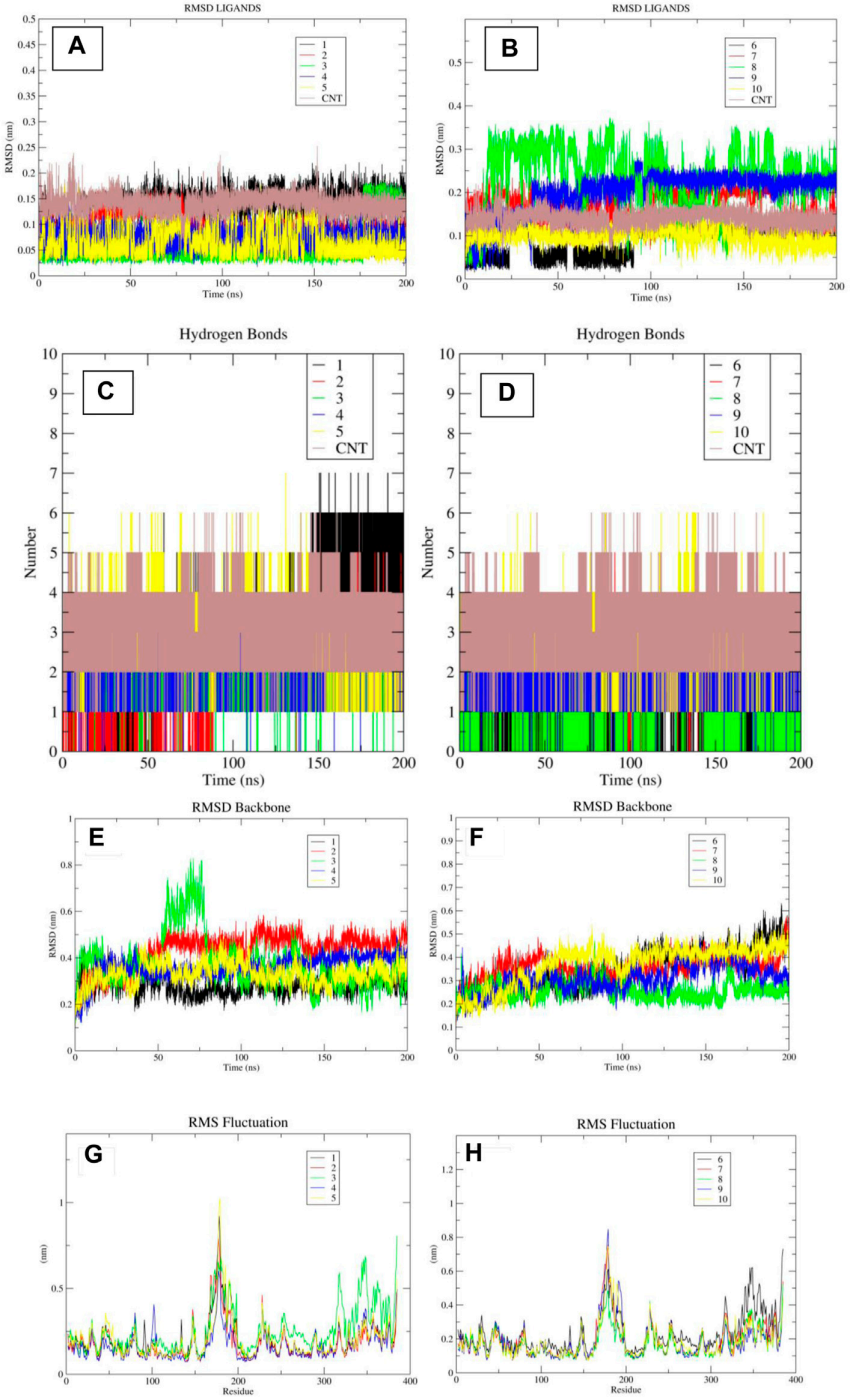
**(A–H)**: The images “**(A, B)**” indicates the RMSD plots of ligands when in bound form with TBK1 protein. The images “**(C, D)**” indicate the hydrogen bond plots of the ligands during the course of simulation. The images “**(E, F)**” indicate the RMSD plot of the protein backbone, while the images “**(G, H)**” indicate the RMSF plot of the protein residues.

MD-based experiments were conducted on further selected compounds using the Gromacs 2022.4 software tool. Upon careful examination of the trajectories of the different molecules (Figures 8A, B), it was seen that a number of compounds on the short list exhibited stable RMSD plots, with RMSD values below 3 Armstrong. All molecules, except for molecule 8, exhibited stable RMSD plots throughout the simulation. Only minor conformational changes were observed at RMSD values below 3 Å, indicating their robust pose stability. Compound 1, 2, 3, 4, 5, 6, and 10 demonstrated stable orientations and exhibited RMSD values that were predominantly in proximity to or lower than the RMSD values of the control molecule. The compounds designated as 7 and 9 likewise achieved a stable conformation, but their RMSD values were slightly higher than that of the control molecule. In contrast, Molecule 8 exhibited consistent deviations as a result of its limited pose stability, consistently undergoing conformational changes during the molecular dynamics simulation. The investigation of hydrogen bond interactions indicated that molecules 1, 4, 5, and 10 exhibited an equivalent or greater number of hydrogen bonds compared to the control molecule (see Figures 8C, D).

The molecules created a total of 17 hydrogen bonds, with 5, 4, 4, and 4 bonds being formed by each separate molecule. The remaining molecules, specifically molecules 2, 3, 6, 7, 8, and 9, exhibited an average number of hydrogen bonds of 3, 3, 2, 3, 1, and 3, respectively. Remarkably, in simulated experiments, it was seen that chemical compound 1 formed a maximum of six hydrogen bonds with the receptor following a minor adjustment in its benzene-1,3-diol moiety at around 150 nanoseconds. The aforementioned findings suggest that this particular group plays a pivotal role in facilitating hydrogen bond interactions with the receptor, hence presenting potential applications in the development of innovative inhibitors. Also, the RMSD plots of the protein backbone and RMSF plot of protein residues indicated that most of the molecules have consistent RMSD in the backbone except for the protein bound to the ligand 3, which displayed highest deviations (Figures 8E–H). The results of the MMPBSA calculations indicated that four compounds had a water-based binding affinity that was comparable to, or even higher than, that of the control molecule. The affinities of the compounds, specifically molecules 1, 4, 5, and 10, were observed to have values of −48.78, −45.47, −46.78, and −47.56, respectively. Based on a meticulous examination of the RMSD plots, as well as an assessment of the hydrogen bonding pattern and the water-based scoring scheme, it is possible to deduce that four specific molecules, namely, 1, 4, 5, and 10, should be given priority for subsequent review. Furthermore, it has been shown that groups such as benzene-1,3-diol and pyrrole have potential for utilization in the development of new inhibitors against the TBK1 inhibitor.

### 3.8 Similarity index studies and physiochemical properties studies

The RDKit module in Python was employed to compute the chemical similarity index using a dataset of known inhibitors published in CHEMBL that exhibit antagonist activity against the TBK1 protein. It was noted that all compounds had a similarity index below 74% when compared to the reported molecules, as measured by both the Tanimoto MACCS value and the similarity index value was found to be below 38% in terms of the Tanimoto MORGAN value. Therefore, all of the compounds possess unique structures that have not been previously documented in relation to the TBK1 protein. During the manual cross-checking process for reported activity of the molecules, a similar observation was made in the “Reported Activity” column of the ZINC database. Therefore, it can be inferred that all of the selected hit molecules possess the potential to function as unique compounds that have not been previously documented to have any activity against the TBK1 protein. Based on previous computational investigations, it is recommended to prioritize these compounds for further *in vitro* studies. The molecular similarity data has been presented and summarized in Table 4. The physiochemical characteristics examination of the molecules revealed that all of them possess a molecular weight that is lower than that of the control molecule BX795, as shown in Table 5. With the exception of molecule 2, all molecules exhibited LogP values within an acceptable range below 5. Log S values were also below 5 for most molecules except 2 and 8. The drug likeness was high for all molecules based on the lipinki’s rule as predicted by the SwissADME web server. The observed count of hydrogen bond donors and acceptors for all compounds was found to be less than 10 and 5, respectively.

**TABLE 4:** The table presents a concise overview of the Tanimoto similarity index for the most highly ranked compounds.

Sr No.	Compound	Tanimoto MACCS similarity	Tanimoto MORGAN similarity	CHEMBL compound
1	1	0.492537	0.369748	CHEMBL2207179
2	2	0.557692	0.371681	CHEMBL3931286
3	3	0.701493	0.319328	CHEMBL3936038
4	4	0.478261	0.33945	CHEMBL3956718
5	5	0.655172	0.315315	CHEMBL3902251
6	6	0.75	0.333333	CHEMBL3900950
7	7	0.611111	0.291667	CHEMBL3928914
8	8	0.7	0.290541	CHEMBL2011937
9	9	0.463768	0.327273	CHEMBL3956718
10	10	0.744681	0.380952	CHEMBL3931286

**TABLE 5:** The table indicates the ADMET profile of the selected 10 molecules.

Name of compound	Molecular weight	Lipinki druglikeness	GI absorption	LogS	LogP	Hydrogen bond donors	Hydrogen bond acceptors
ZINC12113810	**346.140**	Yes	High	−4.72	**3.268**	6	3
ZINC12370930	**432.060**	Yes	High	−6.73	**5.673**	5	2
ZINC04338236	**395.100**	Yes	High	−3.51	**2.100**	9	4
ZINC08548500	**397.080**	Yes	High	−4.84	**4.110**	6	2
ZINC26330579	**404.110**	Yes	Low	−3.99	**3.544**	8	3
ZINC98100095	**350.090**	Yes	High	−3.68	**1.934**	7	2
ZINC89797427	**364.120**	Yes	High	−4.86	**4.144**	6	2
ZINC02278530	**529.070**	Yes	High	−6.43	**4.951**	8	3
ZINC22111451	**417.020**	Yes	High	−5.13	**4.177**	6	2
ZINC69924561	**333.130**	Yes	High	−3.63	**2.105**	8	2
BX795	**591.090**	Yes	Low	−6.00	**4.122**	9	4

## 4 Conclusion

In this study, machine learning techniques were applied to computationally identify novel inhibitors of the TBK1 protein. Utilizing pharmacophore-based screening, we efficiently reduced the search space to 6.59%, resulting in a database of 132,571 entries. Subsequent machine learning-based filtering further reduced the database to 0.66% of its original size, with the optimized model demonstrating exceptional performance. Both the pharmacophore and machine learning model can serve as efficient filtering methods for binary categorization of novel inhibitors from large commercial and non-commercial databases. These methods offer efficient filtration for identifying potential inhibitors from extensive databases, warranting further investigation. The study’s innovative approach, integrating pharmacophore and machine learning methods, fills a gap in the literature concerning virtual screening targeting TBK1. With the identification of four promising compounds, future research will focus on assessing their activity in cell-based or enzyme-based systems, potentially enhancing their therapeutic efficacy against TBK1. Although, two novel groups including benzene-1,3-diol and pyrrole were found, which can be used in novel ligand design to enhance potency of previous scaffolds against the TBK1 protein. The studies here lays the foundation for using AI integrated methodology for screening new inhibitors against the TBK1 protein. This will serve as the focus of our research team’s future endeavors.

## Data Availability

The original contributions presented in the study are included in the article/Supplementary Material, further inquiries can be directed to the corresponding author.
