# Supplementary material for: Identification of TBK1 inhibitors against breast cancer using a computational approach supported by machine learning
Source: Front Pharmacol. 2024 Mar 19;15:1342392. doi: 10.3389/fphar.2024.1342392 (PMC10985244; doi:10.3389/fphar.2024.1342392)
Supplement: Supplementary file 1 [file Table1.DOCX]

Supplementary Data:

**Table s1:** The table indicates the structures, name of compound and code used of the top shortlisted molecules.

| **Sr. No.** | **Name of Compound** | **Code Used** | **Structure** |
| --- | --- | --- | --- |
| 1 | ZINC12113810 | 1 | 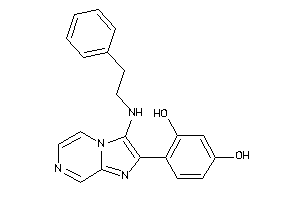 |
| 2 | ZINC12370930 | 2 | 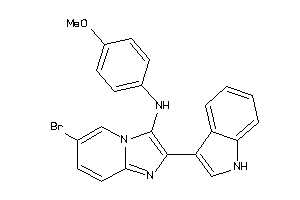 |
| 3 | ZINC04338236 | 3 | 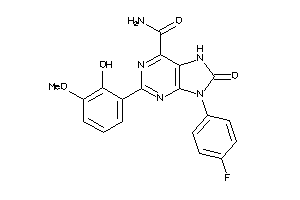 |
| 4 | ZINC08548500 | 4 | 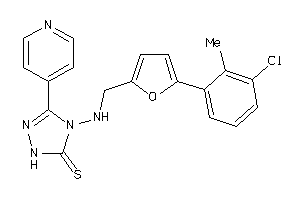 |
| 5 | ZINC26330579 | 5 | 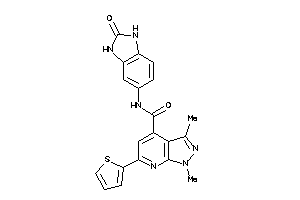 |
| 6 | ZINC98100095 | 6 | 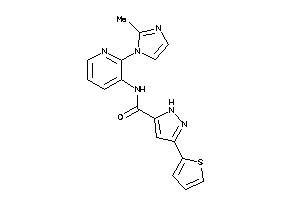 |
| 7 | ZINC89797427 | 7 | 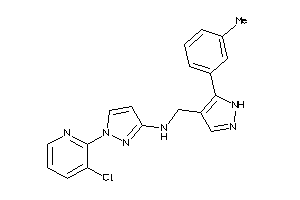 |
| 8 | ZINC02278530 | 8 | 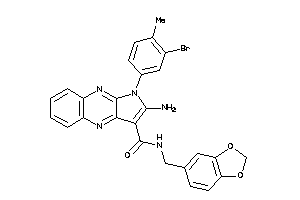 |
| 9 | ZINC22111451 | 9 | 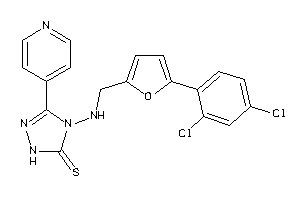 |
| 10 | ZINC69924561 | 10 | 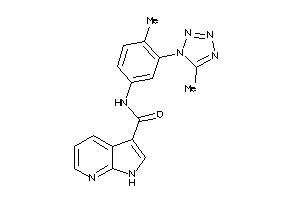 |
| 11 | BX795 | CNT | 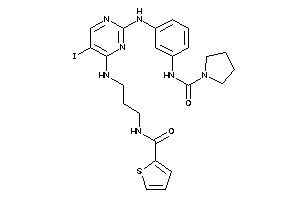 |
